# Where you live matters more than who you know: Context‐level contact as a stronger predictor of post‐war reconciliation than individual‐level contact

**DOI:** 10.1111/bjso.12913

**Published:** 2025-06-12

**Authors:** Sabina Čehajić‐Clancy, Clemens Lindner, Pascal Gelfort, Julia Elad‐Strenger, Thomas Kessler

**Affiliations:** ^1^ Department of Psychology Stockholm University Stockholm Sweden; ^2^ Department of Social Psychology Friedrich Schiller University Jena Jena Germany; ^3^ Department of Political Studies Bar Ilan University Tel Aviv Israel

**Keywords:** conflict resolution, intergroup contact, intergroup relations, reconciliation, social context

## Abstract

Intergroup contact theory and research argue and demonstrate that intergroup relations, even in post‐war societies, could be improved by individuals' positive contact experiences with outgroup members. This study extends this argument by investigating whether post‐war reconciliation is determined by the amount and quality of individuals' *personal contact* experiences with members of former adversary groups (individual‐level contact) *and/or* by the amount and quality of contact that occurs within the context in which they live (*context‐level contact*). Using multilevel analyses among large representative youth samples from ethnic majorities (*N* = 2758) and minorities (*N* = 1751) nested across 40 administrative regions in five post‐war countries (Bosnia and Herzegovina; North Macedonia; Kosova; Montenegro; Serbia), we provide substantive evidence that context‐level contact is a stronger determinant of reconciliation, surpassing the influence of individual‐level contact. Evidence from this research demonstrates the critical influence of the social context, particularly the amount and quality of intergroup contact that occurs within individuals' immediate surroundings, on post‐war reconciliation, and provides important guidelines for policies and interventions fostering positive intergroup relations.

## INTRODUCTION

From the protracted and ongoing violence in the Middle East to current wars in Ukraine and Sudan, conflicts between ‘us’ and ‘them’ abound and rebound around the globe (Deutsch et al., 2011; Hegre & Nygård, 2015; Von Einsiedel et al., 2017). To achieve sustainable conflict resolution and reconciliation, social‐psychological theory and research have identified some effective approaches and strategies for improving intergroup relations (Čehajić‐Clancy & Halperin, 2024; Halperin et al., 2023) through changing attitudes (Cohen‐Chen et al., 2014; Elad‐Strenger et al., 2019; Halperin et al., 2011) and sometimes even behaviours towards members of adversarial groups (Čehajić‐Clancy & Olsson, 2023). One of the most studied social‐psychological approaches for improving intergroup relations, also in contexts of intergroup conflict, has been intergroup contact (Allport et al., 1954; Čehajić & Brown, 2010; Čehajić‐Clancy et al., 2016; Pettigrew & Tropp, 2008). For example, having positive intergroup contact experiences with outgroup members has been shown to be linked to reduced prejudice (Paluck, 2009), increased empathy and perspective‐taking (Cehajic et al., 2008), decreased intergroup anxiety (Tropp, 2015) and increased support for reconciliation (Čehajić & Brown, 2010). Although important, most of the studies examining the effects and correlates of intergroup contact in contexts of conflict (some exceptions include Penić et al., 2021) focused on individuals' personal contact experiences with adversary group members (i.e. individual‐level contact) while neglecting the relevance of intergroup contact occurring in the social context where people live (i.e. context‐level contact). In this paper, we compared the relative effects of context‐level vs. individual‐level contact on reconciliatory attitudes in a largely understudied context: post‐war countries previously affected by grave intergroup violence. We used representative samples of the youth population living in five such countries in the Western Balkan.

### Individual‐level contact

By now, research has firmly established that the frequency and quality of contact individuals have with outgroup members determines individuals' intergroup attitudes and behaviours, even in contexts of real‐world conflicts and wars (Tropp, 2015; Wagner & Hewstone, 2012). Those studies examining contact in conflict or post‐conflict contexts followed the traditional approach used in intergroup contact research, focusing primarily on individuals' personal contact experiences with adversary group members. These studies suggest that individuals who report more positive contact experiences with outgroup members report improved reconciliation‐oriented attitudes and behaviours, including higher trust and forgiveness towards the adversary outgroup (Cehajic et al., 2008; Čehajić‐Clancy & Bilewicz, 2017; Dixon et al., 2010; Kende et al., 2022; Mousa, 2020; Paolini et al., 2004; Tam et al., 2009). For example, the study by Kende et al. (2022) examining attitudes and trust between Greek Cypriots and Turkish Cypriots showed that positive intergroup contact is vital for building trust and positive attitudes, even in ethnically mixed communities with a legacy and experience of conflict.

However, the same line of research has also established that individuals living in contexts affected by conflict also report having negative contact experiences with outgroup members (Paluck et al., 2019; Paolini et al., 2010), which, in turn, can lead to so‐called ‘backfire’ effects. For example, in such conflict‐ridden contexts, superficial forms of contact can reinforce existing negative beliefs about others and thus may lead to confirmation of stereotypes and prejudice, rather than a reduction of prejudice (Paluck et al., 2019; Paolini et al., 2010). Furthermore, intergroup contact that highlights power differences between groups or unresolved historical injustices, common in conflict contexts, may lead to increased resentment and hostility, rather than reconciliation (Paolini et al., 2010), increased negative emotions (Stephan & Stephan, 1985), and even intensification of ingroup identity (Tajfel, 1982).

In addition to these ‘negative’ implications of individual‐level contact in contexts of conflict, other lines of research have established that individual‐level contact can also reduce the urgency of addressing systemic inequalities and thus lead to complacency and reduction in collective action (Cakal et al., 2011; McKeown & Dixon, 2017; Reimer & Sengupta, 2023) rather than empowering disadvantaged or minority groups. Regarding the so called ‘complacency’ effects of contact for minorities, recent studies focusing on ethnic minority group members (Bracegirdle et al., 2023) found no evidence that contact with members of the advantaged outgroup predicts perceived discrimination. Using a social network analysis approach, these studies suggest that perceived discrimination is more strongly shaped by processes of ingroup socialization rather than intergroup contact. Although recent meta‐analyses estimate the effect sizes of individual‐level contact on reducing collective action tendencies to be small and variable (Reimer & Sengupta, 2023), concerns remain about the limitations of individual‐level contact, particularly for members of minority or disadvantaged groups. Furthermore, these issues and concerns are particularly problematic in settings where structural or psychological injustices remain unaddressed (Hässler et al., 2020) such as in conflict‐affected contexts. In conclusion, while individual‐level contact can contribute to positive intergroup outcomes (e.g., improved intergroup attitudes), it also carries risks and potential negative consequences (e.g., reduced collective action). As such, its effects must be evaluated within the broader social context in which it occurs.

### Context‐level contact

Despite their large contribution to the understanding of the effects of individual contact on intergroup relations, most studies on intergroup contact have largely neglected the critical influence of the social context on individuals' attitudes and behaviours (Allport et al., 1954; Blanchard et al., 1991; Pettigrew, 2018; Pettigrew & Hewstone, 2017; Sherif, 1936). By overlooking the impact of crucial contextual features, such as social norms or the amount and quality of intergroup interactions occurring within individuals' surroundings–which significantly influence attitudes and behaviours (Paluck et al., 2019; Pettigrew, 2018), our understanding regarding the effectiveness of intergroup contact in improving intergroup relations will remain limited.

Here, we address this crucial gap by investigating whether living in a context with higher amount and quality of intergroup contact, irrespective of individual's own contact experiences, can predict reconciliation in post‐war societies. The frequency of contact in a social context represents the statistical regularity (‘what most people do’), also referred to as the ‘descriptive norm’ (Cialdini et al., 1991), which has been found to have a strong influence on individuals' own attitudes and behaviour in a given context (Cialdini et al., 1991; Milgram et al., 1969). Following Sherif (1936), individuals adjust their judgements and attitudes to the prevalent judgements and attitudes of others around them, creating what becomes a social reality for individuals within these contexts.

Based on this, we predict that a context with a higher frequency and quality of intergroup contact may indeed provide a favourable descriptive norm of contact (‘intergroup contact is common and positive’), which may enhance individuals' reconciliation tendencies, whereas contexts with a lower frequency and quality of intergroup contact may provide an unfavourable descriptive norm of contact (‘intergroup contact is less common and negative’), which may confirm prejudice and keep reconciliation tendencies low. In particular, frequent and positive intergroup contact at the contextual level may signal that harmonious intergroup interactions are normative, typical and acceptable and thus influence individuals' attitudes and behaviours towards outgroup members. These assumptions adopted in our study align closely with the extended contact hypothesis proposed by Steve Wright et al. (1997), which posits that observing or being aware of positive intergroup relationships involving ingroup members can indeed foster more favourable attitudes towards the outgroup, even without direct personal contact.

Although most research on intergroup contact and reconciliation has focused on individual‐level contact, there is indeed some important evidence that indicates that the amount of contact within the region where people live and interact on a daily basis plays an important role in improving general outgroup evaluations. Specifically, some studies suggest that a person living in a context where other people, on average, have more positive intergroup contact is likely to be less prejudiced than a person with more individual positive contact but living in a context where other people, on average, have less intergroup contact (Christ et al., 2014; Kauff et al., 2016; Wagner et al., 2006). More specifically, Christ et al. (2014) showed, first, in cross‐sectional studies, that living in a social context (e.g., a neighbourhood) in which others have a high amount of positive intergroup contact is associated with more positive intergroup attitudes (for both majority and minority group members), independent of one's own contact experiences. Then, in longitudinal studies, they showed the same contextual effect of contact led to an improvement in intergroup attitudes. In other words, contact at the level of the social context influences individual attitudes over and above contact experienced at the individual level. Studies that have a direct relevance to our work set in post‐war countries is research set in conflict‐affected contexts. For example, recent research by Kende et al. (2022) examining intergroup attitudes between Greek Cypriots and Turkish Cypriots showed that living in a village (as a contextual unit) where others frequently engage in intergroup contact was independently associated with more positive intergroup attitudes and higher levels of trust (even for those who personally had little or no individual‐level contact). These findings suggest that higher levels of contextual contact, even in post‐war countries, might be predictive of more positive intergroup attitudes related to reconciliation.

In contexts of war and large‐scale violence, intergroup relations are particularly strained, inherently characterized by extreme intergroup distrust and social distance due to experienced harm (Cehajic et al., 2008; Čehajić‐Clancy et al., 2016; Penić et al., 2021). Intergroup attitudes in such contexts extend beyond mere negative evaluations of outgroups. In addition to focusing on reconciliation attitudes in post‐war societies, it is also crucial to understand the effects of context‐level contact for both minority and majority group members living in the same context, as both majority and minority perspectives are critical for understanding intergroup relations more broadly (Dixon et al., 2010). Indeed, Christ et al. (2014) demonstrated the importance of contextual contact for predicting intergroup attitudes for both majority and minority group members. Furthermore, research by Kauff et al. (2016) explored how majority members's contact experiences across regional contexts are associated with minority members' support for their ingroup rights. In other words, Kauff and colleagues examined how immigrants' (minority) support for their group's rights is influenced by majority (non‐immigrant) members' intergroup contact experiences in social contexts in which they live. Their findings indicate that minority group members exhibit greater support for anti‐discrimination laws and immigrant rights when residing in social contexts where majority group members report positive intergroup contact experiences (Kauff et al., 2016). This suggests that a favourable intergroup climate, fostered by positive interactions among majority members, can enhance minority members' advocacy for their ingroup rights. The study underscores the significance of the social context in shaping minority groups' attitudes towards their rights. However, what remains unexplored is a direct comparison of both majority and minority group members residing *within* the same context on reconciliation attitudes in post‐conflict contexts.

Studies that have directly compared the effects of contact for both majority and minority group members focused primarily on the effects of individual‐level contact experiences and suggest that minority members' attitudes might be less affected by individual‐level contact experiences than majority group members' attitudes (Binder et al., 2009; Kende et al., 2018; Pettigrew & Tropp, 2008), in part due to an awareness of experienced discrimination (Saguy & Chernyak‐Hai, 2012; Tropp, 2007). However, recent meta‐analyses including studies from diverse social contexts point to reliable associations between individual‐level contact and reduced prejudice, irrespective of the degree of threat and discrimination (Van Assche et al., 2023). Nevertheless, to date, no studies have explored context‐level contact effects for both minority and majority members living and residing within the same social context challenged by a history of intergroup conflict. In conclusion, our research seeks to address two questions. First, can we expect context‐level contact effects to extend beyond prejudice reduction and predict improved attitudes related to post‐war reconciliation over and beyond personal contact experiences? Second, can we expect this context‐level effect to hold for both ethnic majority and minority group members residing *within* the same social context?

### Study context

To answer these questions, we tested the effects of individual‐level and context‐level contact on inter‐ethnic reconciliatory attitudes among different ethnic groups in the Western Balkan region across five countries (Bosnia‐Herzegovina, Serbia, Montenegro, North Macedonia, Kosovo) affected by the conflict during the 1990s. The conflict in the Western Balkans during the early 1990s emerged from the disintegration of the former Socialist Federal Republic of Yugoslavia (SFRY) and was primarily driven by the rise in ethnic nationalism, particularly in Serbia under Slobodan Milošević (Becirevic, 2014). The wars that emerged across the Western Balkans were characterized by deep‐seated ethnic divisions and grievances. These conflicts were fuelled by historical narratives of victimization, religious differences and competition for territorial control. Grave breaches of international humanitarian law occurred, including ethnic cleansing and acts of genocide (Becirevic, 2014), which resulted in the deaths of civilians, displacement of millions and significant trauma across the region. They shattered communities, leaving deep scars and enduring intergroup tensions that continue to influence political and social dynamics in the region today (Shared Futures: Youth Perceptions on Peace in Western Balkans, 2021). Although the extent of physical destruction and psychological harm varied significantly across Western Balkan countries, interethnic relations throughout the region remain marked by deep distrust, high social distance and low levels of forgiveness (Castano et al., 2024; Cehajic et al., 2008; Shared Futures: Youth Perceptions on Peace in Western Balkans, 2021). Furthermore, the situation in the entire region remains volatile and politically unstable. Thus, investigating and identifying effective approaches towards improving intergroup relations remains an important question in this wider Western Balkan context.

Given that our study focused on perspectives of both ethnic majorities and ethnic minorities spread across and within each country, it is important to note that the ethnic composition of each country (where we collected data) differs. In other words, the ethnic groups that constitute a majority or a minority vary across countries and regions within each country. For example, looking at Bosnia and Herzegovina as a country, three ethnic groups (Bosniaks, Serbs and Croats) officially constitute so‐called constituent ethnic majority. However, looking at specific regions *within* Bosnia and Herzegovina, some of those ethnic groups constitute a statistical majority, while other ethnic groups constitute a statistical minority. For example, Bosniaks constitute an ethnic majority in the capital city of Sarajevo, while, for example, Serbs who live in Sarajevo constitute an ethnic minority. On the other hand, looking at Banja Luka city (another major city in Bosnia and Herzegovina), Serbs constitute an ethnic majority, whereas Bosniaks constitute an ethnic minority. In conclusion, the ethnic majority/minority status is not fixed at the level of ethnicity or country and depends on the region where people live.

### Current study

In our research, we operationalized context‐level contact as the average amount and quality of intergroup contact that actually occurs within the administrative region where people live and interact on a daily basis. In other words, context‐level contact refers to actual direct interactions that occur within the everyday environments where people live, work, and socialize (defined as regions). Unlike extended intergroup contact, which refers to knowledge about intergroup interactions (Wright et al., 1997), context‐level contact is characterized by actual real‐life interactions occurring in shared spaces (Christ et al., 2014). Social context or communities in which people live and interact on a daily basis were defined as administrative regions with public services utilized by people on a daily basis. Hence, context‐level contact reflects the mean contact for each region (40 administrative divisions/units nested in five countries) which is used as a predictor for the dependent variables at the individual level for individuals living within the respective region. For more detailed information on specifics regarding regions in each respective country, please refer to the Supporting Information (Tables S2–S6).

In order to approximate reality and capture the actual amounts of contact, which occur in peoples' surroundings, we obtained data from large representative samples of youth from post‐war countries. Following the United Nations Security Council Resolution calling for an increased focus on the young generation living in post‐war countries as key ‘carriers’ of peacebuilding efforts (United Nations Security Council Resolution 2250 on Youth, Peace and Security, 2015), we deliberately focused on collecting data from youth. As our samples are representative of the youth population living in those five countries, the average amount of contact in a region can be regarded as an adequate indicator of the actual contact frequency and quality among the youth population in that respective region.

Furthermore, we accounted for the possibility that post‐war contexts could exacerbate the role of minority status and thus increase experiences of more negative intergroup encounters (Saguy & Chernyak‐Hai, 2012). Hence, we empirically captured both contact quantity (‘In your area, how many people from other ethnic groups do you know, at least as acquaintances?’) as well as contact quality (‘In your area, how many people from other ethnic groups do you know that you would consider to be friends?’) at both individual and contextual levels. Based on the literature reviewed, we hypothesized that where people live will be a stronger predictor of inter‐ethnic reconciliation in comparison to who they personally know or interact with. Furthermore, we examined whether the effects of context‐level contact will be robust enough to be effective for both ethnic majorities and minorities living in the same region. As stated in the section on the study context, the ethnicity of our participants was operationalized using a self‐report measure in which participants indicated their ethnic group in the specific administrative region where they live. Furthermore, all of our analyses controlled for important confounding variables at both levels, which included ethnic fractionalization—index of ethnic diversity of the region in which people live representing contact opportunity (Fearon, 2003), country at the contextual level, urbanity vs. rurality of the community, participants' gender and education level, mobility (extent to which participants have travelled outside the Western Balkans in the past 2 years), age, ethnicity and personal experience with ethnic‐based discrimination at the individual level. These control variables were selected on conceptual and empirical grounds, validating critical socio‐demographic correlates of intergroup relations in general and post‐conflict reconciliation in particular (Shared Futures: Youth Perceptions on Peace in Western Balkans, 2021).

## METHODOLOGY

### Sample and procedure

Data were collected using an in‐person survey method from youth representative samples in Bosnia‐Herzegovina, Serbia, Montenegro, North Macedonia, Kosovo (*N* = 4509) during 2020, by the United Nations Development Program (UNDP) Office in Albania and Regional Youth Cooperation Office (RYCO) from Western Balkan countries. Data for all five samples are representative of 15–29‐year‐old members of the ‘post‐war generation’ in the Western Balkans and included respondents of diverse ethnicities, educational and socio‐economic backgrounds as well as youth living in urban and rural areas. All five studies were conducted following the Declaration of Helsinki and under the direct supervision of the UNDP office in Albania. All participants provided informed consent. For minors, a written consent of the parent or guardian was obtained. Following consultations with the ethics board, authors' institutions did not require review of the study and consequently, no ethical approval was sought. Questions were developed in consultation with a youth advisory group of 23 young women and men across the Western Balkans region.

As representative samples, data for all five studies included respondents of diverse ethnicities, educational and socio‐economic backgrounds as well as youth living in urban and rural areas. When selecting the primary sampling units, stratification and probability was applied. Households were selected via the random route method, and the sample distribution reflected official data in the region as pertains to gender and age parameters. About 20% of the young people interviewed reported not to be in education, employment or training, and 40% reported living in households with a modest or poor economic situation. The data collection placed a special focus on representativeness of ethnicities in the Western Balkan region. Ethnicity was ascertained through the self‐identification of the respondent. When needed, boost samples were applied. Boost samples were not applied when the various ethnicities were captured sufficiently in the main sample (see Table 1).

**TABLE 1:** Overview of sample sizes.

Country	Majority (*n*)	Minority (*n*)	Total (*n*)
Bosnia and Herzegovina	11% (488)	10% (435)	20% (923)
North Macedonia	13% (587)	7% (309)	20% (896)
Kosova	14% (650)	5% (244)	20% (894)
Montenegro	8% (382)	11% (514)	20% (896)
Serbia	14% (651)	6% (249)	20% (900)
Total	61% (2758)	39% (1751)	100% (4509)

The methodology used for the collection of the data was in‐person Computer‐Assisted Personal Interviews, whereby the interviewer used an electronic device to record the responses. Data were collected by survey agencies in respective countries directly, contracted by the UNDP Office. The research team was not involved in the data collection process other than providing consultations and guidance for sample selections, questionnaire design, and identifying pre‐ and post‐selection criteria in direct communication with the UNDP Office and survey agencies. Interviews with more than 30 percent of empty or non‐valid responses (such as *do not know* or *refuse to answer*) were considered unsuccessful and excluded. The average missing rate (variables with missingness/all variables assessed) for the complete data set with all variables assessed in the questionnaire was 6.46% (*SD* = 2.02 [3.39–24.46]). For the variables included in our analyses only, the average missing rate was 1.61% (*SD* = 4.05 [0–41.94]). Two cases (0.04% of the total sample) had more than 30% missing data across the measured variables. Given such low rates of missing data, we did not use imputation or exclude data with higher rates of missingness to avoid bias. The survey was prepared in English, translated into local languages (Bosnian, Macedonian, Montenegrin and Serbian), and back‐translated for quality assurance.

During and following the data entry phase, the data were subject to five kinds of checks: range checks, checks against reference data, skip checks, consistency checks and typographic checks. The quality of the data was controlled for the first few interviews by each interviewer and through 10%–15% random backchecks of each interviewer's completed interviews. An additional 10 percent of randomly selected questionnaires were checked through a follow‐up with the respondent via telephone/mobile. Upon collection, data checks and entry, all data were anonymized. The research team did not have access to any personal or identifying information of the respondents at any point. For further information, an anonymized link to raw data, data analyses, models and plots has been made available. https://osf.io/2r5s8/?view_only=63e2c765cdf146be8e4a2fdc132f261d.

### Survey questions

Questions were developed in consultation with a youth advisory group of 23 young women and men across the Western Balkans region. The advisory group was put together through an open call and included students, young activists and young professionals engaged in various peacebuilding and community work, ranging from advocacy and academic efforts to grassroots activities. The co‐design process consisted of seven online consultations with the youth advisory group.

*Contact quantity* was measured with the following item: ‘In your area, how many people from other ethnic groups do you know, at least as acquaintances?’ Participants could choose one of the following options: none, very few, some, many.

*Contact quality* measure aimed to capture the amount of outgroup friends participants report to have ‘In your area, how many people from other ethnic groups do you know that you would consider to be friends?’ Participants could choose one of the following options: none, very few, some, many.

Reconciliatory attitudes included the following scales:

*Forgiveness* was measured with the following item: Thinking about the past of your society and the past of the Western Balkan region, to what extent do you agree or disagree with the following statement ‘I am willing to forgive other groups for what they did to my people in the past’. Participants answered on a 5‐point scale ranging from 1 (strongly disagree) to 5 (strongly agree). They were also given an opportunity not to answer.

*Trust* was measured with the following item: ‘I trust most peoples of the Western Balkans despite events that may have occurred in the past’. Participants answered on a 5‐point scale ranging from 1 (strongly disagree) to 5 (strongly agree). They were also given an opportunity not to answer the question.

*Social Distance* was measured with four questions about the people participants might be willing to marry. If you were to choose now who to marry, evaluate how difficult the following scenarios would be to marry: ‘Someone who belongs to a different religion’; ‘Someone who has a different outlook on religion’; ‘Someone from my country who belongs to a different community’; ‘Someone from elsewhere in the Western Balkans who belongs to a different community’. Participants answered on a 5‐point scale ranging from 1 (not at all difficult) to 5 (extremely difficult). They were also given an opportunity not to answer. The reliability of this scale was highly satisfactory with *Cronbach's alpha* = .87.

*Approach Behaviour* was measured with four items aimed to capture different degrees of approach oriented behaviours towards the most distant ethnic outgroup in the context of Western Balkans: Thinking of the group in the Western Balkan region that in your view are most distant from your people, to what extent do you agree or disagree with the following: ‘I am willing to have members of this group as my neighbours’; ‘I am willing to have them as colleagues or school mates’; ‘I am willing to have them as close friends’; ‘I am willing to marry, or have people in my family marry, members of this group’. Participants answered on a 5‐point scale ranging from 1 (strongly disagree) to 5 (strongly agree). They were also given an opportunity not to answer the question. The reliability of this scale was highly satisfactory with *Cronbach's α* = .89.

*Control variables* included two context‐level control variables: participants' settlement type (urban/city vs. rural/village) and ethnic diversity of the community (ethnic fractionalization; Fearon, 2003) and seven individual‐level control variables: gender, age, ethnicity, education, mobility (degree of travel experience outside their own country), civic engagement (degree of civic engagement in the last 12 months) and experience with ethnic‐based discrimination. Across all questions, participants were given the opportunity not to answer.

### Analytic strategy

In order to compare and test the effects of context‐level contact (i.e., testing whether there is a significant difference between the context‐level and individual‐level effect of contact), we followed the analytic approach suggested by Christ et al. (2014) which contains analysing and reporting both the within‐ and between‐level effects and, importantly, the difference between the two indicating the contextual effect. In addition to calculating the contextual effect, we also calculated and reported the effect sizes of the estimated contextual effect. Furthermore, in order to control for differences in the reliability of all measures on the individual and context level, we used multilevel structural equation modelling with latent variables for independent as well as dependent variables on both levels where respondents (individual level) were nested within regions (context level) as suggested by Christ et al. (2017). Given the small number of countries, country as a potential Level 3 predictor was eliminated (Bryan & Jenkins, 2016) but included as a level 2 control variable to account for possible differences between countries. As stated above, we applied a multilevel structural equation modelling (MSEM) approach with latent variables, as studies have shown that aggregating manifest variables at the contextual level can lead to substantially biased estimates of context‐level effects (Lüdtke et al., 2008). By modelling latent instead of manifest variables, MSEM accounts for the measurement error, resulting in less biased estimates of the contextual effect (Christ et al., 2017). Latent individual‐level contact was measured by two single‐item measures that assessed separately contact quality and contact quantity (*r* = .77; *α* = .87). Individual‐level contact measures were centred within clusters (CWC) as individual contact is dependent on cluster membership, that is, the regions in which people live and interact on a daily basis (Enders, 2013). Furthermore, using CWC allows a strict test of relations between level 1 variables, which are of substantive interest to investigate the differential influence of individual‐level and context‐level variables on the dependent variables (Enders & Tofighi, 2007). Consequently, latent context‐level contact was measured by introducing the two individual‐level contact measures (contact quality, contact quantity) at the contextual level of the model.

Given that members of minority and majority ethnic groups may have different experiences or perceive the same intergroup interactions differently, we calculated the contextual contact effects for majorities and minorities separately. In other words, we calculated the effects for each sub‐sample where contextual contact of minority members within the region acts as the predictor of our dependent variables among minority group members and the contextual effect of contact of majority members within the region acts as the predictor of our dependent variables among majority group members.

Finally, and as indicated above, in our analyses, we controlled for important context‐ and individual‐level control variables (Shared Futures: Youth Perceptions on Peace in Western Balkans, 2021) including an index of ethnic diversity (ethnic fractionalization) of regions where people live. Ethnic fractionalization representing the probability of an encounter of two random individuals from different ethnic groups living in the same region was computed using the formula $F\equiv 1-\sum_{i=1}^{n}p_{i}^{2}$ with *p*_1_, *p*_2_, *p*_3_, …, *p*_*n*_ being the population share of an ethnic group (Fearon, 2003). Such an index of ethnic fractionalization or degree of ethnic diversity within a given region provided us with an important and objective correlate of intergroup contact opportunities (Stolle et al., 2008; Sturgis et al., 2011; Van der Meer & Tolsma, 2014). This way, we were able to empirically investigate and differentiate between actual opportunities for contact in one's immediate social context (ethnic fractionalization index) and quantity and quality of intergroup contact, which actually occurs in one's context (context‐level contact).

## RESULTS

Table 2 shows unstandardized estimates for within‐level and between‐level effects of contact on reconciliatory attitudes and behavioural intentions. Importantly, the contextual effect (as the difference between the two levels) including the effect sizes is reported. Results revealed that individuals' approach, social distance, forgiveness and trust tendencies towards adversary ethnic outgroups are more robustly predicted by context‐level contact (between‐level effect) than by personal contact experiences (within‐level contact). Importantly, computed contextual effect across all dependent variables is robust and significant. In other words, the amount and quality of contact in the context in which individuals live predicts participants' personal reconciliation attitudes and behavioural intentions more than who they personally interact with.

**TABLE 2:** Unstandardized estimates for all DVs controlling for participants' sex, mobility, engagement, education, experienced discrimination, age and ethnicity at the individual level and for the urbanity and ethnic fractionalization of the region, including the country at the contextual level.

	Approach			Social distance			Forgiveness			Trust		
	*b* (*SE*)	CI	*p*	*b* (*SE*)	CI	*p*	*b* (*SE*)	CI	*p*	*b* (*SE*)	CI	*p*
Within‐level effect	0.232 (0.022)	[0.19–0.28]	<.001	−0.255 (0.026)	[−0.31 to −0.20]	<.001	0.152 (0.024)	[0.10–0.20]	<.001	0.098 (0.023)	[0.05–0.14]	<.001
Between‐level effect	0.938 (0.126)	[0.69–1.19]	<.001	−0.669 (0.133)	[−0.93 to −0.41]	<.001	0.663 (0.133)	[0.40–0.92]	<.001	0.521 (0.139)	[0.25–0.79]	<.001
Contextual effect	0.706 (0.128)	[0.46–0.96]	<.001	−0.414 (0.136)	[−0.68 to −0.15]	.002	0.511 (0.135)	[0.25–0.78]	<.001	0.423 (0.141)	[0.15–0.70]	.003
Effect size of contextual effect	0.768 (0.151)	[0.47–1.06]	<.001	−0.376 (0.124)	[−0.62 to −0.13]	.002	0.492 (0.133)	[0.23–0.75]	<.001	0.444 (0.149)	[0.15–0.74]	.003
Contextual effect without controls	0.612 (0.122)	[0.37–0.85]	<.001	−0.415 (0.147)	[−0.70 to −0.13]	.005	0.517 (0.137)	[0.25–0.78]	<.001	0.369 (0.137)	[0.10–0.64]	.01
**Variance decomposition**	**Estimate**			**Estimate**			**Estimate**			**Estimate**		
Level 1	0.856			1.306			1.018			0.944		
Level 2	0.261			0.278			0.215			0.185		

Examining the contextual contact effect for majority vs. minority sub‐sample, the results indicate that the robustness of contextual contact of ethnic majorities consistently and robustly predicted all outcome variables among ethnic majorities (see Table 3). On the other hand, the contextual contact of ethnic minorities significantly predicted social distance and approach tendencies, whereas forgiveness and trust were not reliably predicted by contextual contact of ethnic minorities (see Table 3).

**TABLE 3:** Unstandardized estimates for all DVs controlling for participants' sex, mobility, engagement, education, experienced discrimination, age, and ethnicity at the individual level and for the urbanity and ethnic fractionalization of the region, including country at the contextual level for majorities and minorities.

	Approach						Social distance					
	Majority			Minority			Majority			Minority		
	*b* (*SE*)	CI	*p*	*b* (*SE*)	CI	*p*	*b* (*SE*)	CI	*p*	*b* (*SE*)	CI	*p*
Within‐level effect	0.245 (0.029)	[0.19–0.30]	<.001	0.153 (0.041)	[0.07–0.23]	<.001	−0.207 (0.034)	[−0.27 to −0.14]	<.001	−0.357 (0.048)	[−0.45 to −0.26]	<.001
Between‐level effect	0.68 (0.138)	[0.41–0.95]	<.001	0.75 (0.137)	[0.48–1.02]	<.001	−0.376 (0.109)	[−0.59 to −0.16]	<.001	−0.796 (0.198)	[−1.19 to −0.41]	<.001
Contextual effect	0.436 (0.141)	[0.16–0.71]	.002	0.598 (0.143)	[0.32–0.88]	<.001	−0.169 (0.115)	[−0.39 to 0.06]	.14	−0.439 (0.205)	[−0.84 to −0.04]	.03
Effect size of contextual effect	0.563 (0.191)	[0.19–0.94]	.003	0.632 (0.151)	[0.34–0.93]	<.001	−0.19 (0.129)	[−0.44 to 0.06]	.14	−0.391 (0.176)	[−0.73 to −0.05]	.03
Contextual effect without controls	0.603 (0.139)	[0.33–0.88]	<.001	0.517 (0.146)	[0.23–0.80]	<.001	−0.401 (0.134)	[−0.66 to −0.14]	.003	−0.365 (0.227)	[−0.81 to 0.08]	.11
**Variance decomposition**	**Estimate**			**Estimate**			**Estimate**			**Estimate**		
Level 1	0.794			0.885			1.255			1.291		
Level 2	0.348			0.213			0.257			0.437		

	Forgiveness						Trust					
	Majority			Minority			Majority			Minority		
	*b* (*SE*)	CI	*p*	*b* (*SE*)	CI	*p*	*b* (*SE*)	CI	*p*	*b* (*SE*)	CI	*p*
Within‐level effect	0.113 (0.031)	[0.05–0.17]	<.001	0.13 (0.044)	[0.04 to 0.22]	.003	0.084 (0.031)	[0.02–0.14]	.01	0.072 (0.042)	[−0.01 to 0.15]	.08
Between‐level effect	0.678 (0.108)	[0.47–0.89]	<.001	0.32 (0.158)	[0.01 to 0.63]	.04	0.473 (0.119)	[0.24–0.71]	<.001	0.289 (0.17)	[−0.04 to 0.62]	.09
Contextual effect	0.564 (0.113)	[0.34–0.79]	<.001	0.19 (0.165)	[−0.13 to 0.51]	.25	0.389 (0.123)	[0.15–0.63]	.002	0.216 (0.176)	[−0.13 to 0.56]	.22
Effect size of contextual effect	0.635 (0.139)	[0.36–0.91]	<.001	0.169 (0.148)	[−0.12 to 0.46]	.25	0.457 (0.148)	[0.17–0.75]	.002	0.241 (0.181)	[−0.11 to 0.6]	.18
Contextual effect without controls	0.749 (0.136)	[0.48–1.01]	<.001	0.189 (0.163)	[−0.13 to 0.51]	.25	0.434 (0.133)	[0.17–0.69]	.001	0.254 (0.174)	[−0.09 to 0.59]	.14
**Variance decomposition**	**Estimate**			**Estimate**			**Estimate**			**Estimate**		
Level 1	0.992			0.993			0.95			0.892		
Level 2	0.314			0.13			0.204			0.181		

Finally and regarding the role of control variables, the effects of contextual contact remained consistent with and without controlling for relevant individual (e.g. sex, education, etc.) and contextual (e.g., ethnic diversity of the region) variables. For more detailed information on correlations between all measured variables including estimations of standardized estimates for all dependent variables, please refer to the tables in the Supporting Information document.^1^

## DISCUSSION

Data from five countries recently affected by war and crimes against humanity (Silber & Little, 1996) consistently show that a range of individuals' attitudes and behaviours related to inter‐ethnic reconciliation (i.e., forgiveness, trust, social distance, approach behaviours) are robustly predicted by the quantity and quality of actual contact that occurs in participants' social contexts and less so by the quantity and quality of their personal contact experiences (Cehajic et al., 2008; Čehajić‐Clancy & Bilewicz, 2017; Dixon et al., 2010; Hewstone et al., 2006; Mousa, 2020; Paolini et al., 2010; Tam et al., 2009). The degree of ethnic diversity of the communities in which people live (defined as administrative regions in this study) did not predict reconciliation‐related attitudes or behavioural tendencies. However, actual intergroup contact occurring within regions where people live consistently and robustly predicted inter‐ethnic reconciliation for both ethnic majority and ethnic minority perspectives. In other words, the predictive strength of personal contact experiences on inter‐ethnic reconciliation was weaker in comparison to between‐level or contextual contact effects.

These findings are important for at least two reasons. First, they add important insights to the research on a highly vexed relationship between ethnic diversity and social cohesion (Van der Meer & Tolsma, 2014) by providing substantive support for the importance of what is common and what actually occurs within socially diverse communities (Stolle et al., 2008; Sturgis et al., 2011). While ethnically diverse contexts offer opportunities for inter‐ethnic contact, these opportunities alone may not be sufficient for fostering improved intergroup relations. Indeed, Van der Meer and Tolsma (2014) emphasize that diversity alone does not automatically lead to stronger social bonds. In fact, without supportive norms and conditions, diversity can sometimes exacerbate tensions. The research by Stolle et al. (2008) and Sturgis et al. (2011) adds depth to this understanding by demonstrating that when positive intergroup contact is perceived as common and normal within a community, it can help bridge divides and foster better intergroup relations. Indeed, as indicated by our results, having opportunities for intergroup interactions is not sufficient. These contact opportunities need to be transformed into actual frequent and positive intergroup interactions in one's social context (contextual contact) in order to yield improved intergroup relations.

Second, our findings advance theory and research on the effects of intergroup contact on prejudice (Christ et al., 2014) to post‐war reconciliation in important ways by demonstrating that, irrespective of one's own personal intergroup contact experiences, the prospect of intergroup reconciliation can be determined depending on the amount and quality of actual contact, which occurs in the context where people live on a daily basis. As indicated by our results, within‐level contact effects, in contrast, were systematically weaker for predicting intergroup reconciliation indicators, further emphasizing the critical importance of context‐level processes for post‐conflict reconciliation and peace‐building. Prior research, due to its primary focus on the interpersonal nature of contact, has relatively neglected the crucial impact of the contextual contact. Notably, even studies focusing exclusively on individual‐level contact have begun to question the assumption that changes in outgroup attitudes result directly from changes occurring within the individual due to contact experiences. For example, recent findings suggest that the positive effects of individual‐level contact on outgroup attitudes might not be due to within‐person changes occurring due to contact experiences (within‐person effect) but rather due to more stable individual or contextual differences (between‐person effect) suggesting that intergroup interactions at the individual level alone may not be sufficient to improve intergroup relations (Friehs et al., 2024) or promote intergroup equality over time (Sengupta et al., 2023). This distinction between within‐ and between‐person effects is significant, as it implies that personal intergroup interactions alone may be insufficient to drive meaningful improvements in intergroup relations or to promote long‐term social equality (Sengupta et al., 2023). Instead, it highlights the importance of considering the broader social and structural contexts in which contact occurs.

Indeed, our research provides additional evidence for the importance of contextual‐level processes when thinking about post‐conflict and peace‐building processes. Positive changes can be unlocked by creating communities with more frequent and positive intergroup interactions. Low frequency and low quality of contextual contact, with the associated distrust, perceptions of threat and trauma ingrained by war may make reconciliation all the more challenging. Our results show that increased frequency and quality of inter‐ethnic contact, which occurs and takes place in one's immediate social context, are particularly important in these settings and that post‐conflict reconstruction of intergroup relations is primarily a function of where one lives and not necessarily who one interacts with. These frequent and positive intergroup interactions, which occur in one's immediate social context, help to rebuild intergroup relations, suggesting that the success of post‐conflict reconstruction is heavily influenced by the social context in which individuals reside, rather than solely by the specific individuals they interact with. This emphasizes the importance of creating environments that foster regular and positive intergroup contact as a foundation for lasting peace and reconciliation.

Finally, and building upon Kauff et al. (2016), who examined context‐level effects on minorities' attitudes towards majorities, our findings indicate that the effects of contextual contact on social distance and approach intentions were robust enough such that they were not diluted for ethnic minorities living in the same context as majority group members. The contextual contact effects on trust and forgiveness were significantly only for ethnic majorities. These results seem to indicate that all individuals, irrespective of their status and personal contact experiences, may benefit from living in a community where other members engage in intergroup contact. Indeed, our findings suggest that it is not ethnic diversity per se, but rather the amount and quality of ethnic interactions occurring within local communities that can shield wider communities from future conflict and resurgence of ethnic violence through supporting important reconciliation processes.

An important question that remains unanswered and should be addressed in future research is the question of why context‐level contact is powerful for predicting intergroup attitudes, even in post‐war contexts. Furthermore, and considering the correlational nature of our data, the assumed causal path of context‐level contact affecting attitudes and behaviours at the individual level ought to be further examined. To this end, we hypothesize that the impact of context‐level contact on individual‐level attitudes and behaviours might be understood through the lens of the extended contact hypothesis developed by Wright and colleagues (Wright et al., 1997) suggesting that individuals' attitudes towards outgroups can be positively influenced by learning that members of their ingroup have friends or positive relationships with members of the outgroup, even in the absence of direct personal contact. In other words, observing others in one's ingroup engaging positively with outgroup members may explain the effects of contextual contact on individual‐level attitudes and behaviours. Second, the effects may also operate through normative processes in that social contexts where intergroup contact is frequent and positive may lead to the establishment of clear descriptive norms of intergroup harmony. Individuals in such environments may be more likely to perceive that intergroup interactions are common, accepted, and beneficial, which can improve attitudes towards the outgroup. According to Christ et al. (2014), these shared norms can influence even those who may not directly experience intergroup contact themselves. Finally, and as suggested by Christ et al. (2014) and supported by the empirical evidence from the individual‐level contact literature (Pettigrew & Tropp, 2008) living in a context where intergroup contact is frequent and positive may reduce intergroup anxiety for individuals, even if they do not directly engage in such contact. Observing or being aware of positive intergroup relationships (Wright et al., 1997) can diminish intergroup anxiety and perceptions of threat, making individuals more open and inclusive to outgroup members. Even though manipulating real‐life social context might not be feasible and thus limit our ability to establish causal relationships, future research should aim at understanding the mechanisms behind the contextual contact effect on individual attitudes and behaviours.

In conclusion, our findings, while correlational, highlight the important influence of social context on intergroup relations, even in the complex dynamics of post‐war societies (Blanchard et al., 1991; Sherif, 1936). Over time, fostering more positive contact within social context is not only important for regulating key conflict‐related attitudes and behaviours (Hayward et al., 2017; Paluck, 2009; Tropp & Pettigrew, 2005) but, as demonstrated by our results, might be crucial for post‐war reconciliation.

Efforts to support the integration of ethnically mixed communities, where individuals regularly engage in meaningful and positive intergroup interactions, may be vital for preventing future conflict and fostering reconciled intergroup relations. These results also offer important implications for research and practice on psychological intergroup interventions aimed at improving intergroup relations in conflict‐affected contexts (Halperin et al., 2023). Specifically, our findings provide empirical support for the value of adopting a contextual lens when designing and implementing intergroup interventions, emphasizing the need to tailor such efforts to the everyday social environments in which people live (Čehajić‐Clancy & Halperin, 2024).

## AUTHOR CONTRIBUTIONS

**Sabina Čehajić‐Clancy:** Conceptualization; methodology; investigation; validation; supervision; funding acquisition; project administration; writing – original draft; writing – review and editing. **Clemens Lindner:** Software; data curation; formal analysis; visualization; writing – review and editing. **Pascal Gelfort:** Software; data curation; formal analysis; visualization; writing – review and editing. **Julia Elad‐Strenger:** Writing – review and editing; writing – original draft. **Thomas Kessler:** Writing – original draft; conceptualization; writing – review and editing.

## CONFLICT OF INTEREST STATEMENT

The authors declare no competing interests.

## Supporting information


Table S1.–S8.

## Data Availability

An anonymized link to raw data, data analyses including codes, models and plots, has been made public via OSF. The link is provided in the main text file.

## Footnotes

^1^
As it can be seen from additional information provided in the Supporting Information section, there are six regions where the number of majorities or minorities equals 0. We have re‐analysed our data without those six regions and can report that the results remain unchanged.
