# Supplementary material for: Where you live matters more than who you know: Context‐level contact as a stronger predictor of post‐war reconciliation than individual‐level contact
Source: Br J Soc Psychol. 2025 Jun 12;64(3):e12913. doi: 10.1111/bjso.12913 (PMC12163302; doi:10.1111/bjso.12913)
Supplement: Supplementary file 1 — Table S1.–S8. [file BJSO-64-0-s001.docx]

**Supplementary Material**

**Table S1.** Correlation coefficients between all variables.

|  | *Variable* | *1* | *2* | *3* | *4* | *5* | *6* | *7* | *8* | *9* | *10* | *11* | *12* | *13* |
| --- | --- | --- | --- | --- | --- | --- | --- | --- | --- | --- | --- | --- | --- | --- |
| **Predictors** | | | | | | | | | | | | | | |
| 1 | Contact_Individual_ |  |  |  |  |  |  |  |  |  |  |  |  |  |
| 2 | Contact_Context_ | .47*** |  |  |  |  |  |  |  |  |  |  |  |  |
| **Outcomes** | | | | | | | | | | | | | | |
| 3 | Approach | .34*** | .36*** |  |  |  |  |  |  |  |  |  |  |  |
| 4 | Social Distance | -.27*** | -.22*** | -.38*** |  |  |  |  |  |  |  |  |  |  |
| 5 | Forgiveness | .24*** | .29*** | .41*** | -.25*** |  |  |  |  |  |  |  |  |  |
| 6 | Trust | .17*** | .21*** | .38*** | -.20*** | .38*** |  |  |  |  |  |  |  |  |
| **Controls** | | | | | | | | | | | | | | |
| 7 | Ethnic  fractionalization | .14*** | .30*** | .10*** | .02 | .12*** | .024 |  |  |  |  |  |  |  |
| 8 | Age | .079*** | -.0038 | -.0022 | .016 | .007 | -.0025 | -.031* |  |  |  |  |  |  |
| 9 | Gender^a^ | -.054*** | -.003 | -.0081 | .089*** | .0083 | .044** | -.0064 | -.02 |  |  |  |  |  |
| 10 | Mobility^b^ | .18*** | .17*** | .14*** | -.13*** | .066*** | .051*** | .0018 | .13*** | -.029 |  |  |  |  |
| 11 | Urbanity^c^ | .15*** | .075*** | .084*** | -.097*** | .022 | .034* | .048** | -.0042 | -.0015 | .10*** |  |  |  |
| 12 | Engagement^b^ | .16*** | .072*** | .12*** | -.10*** | .073*** | .049** | .12*** | .053*** | -.034* | .17*** | .045** |  |  |
| 13 | Education^d^ | .069*** | -.018 | .032* | .00094 | .014 | .0057 | -.03* | .60*** | .034* | .20*** | .019 | .13*** |  |
| 14 | Experience of  ethnic discrimination^b^ | .13*** | .02 | -.00018 | .0021 | -.00014 | -.031* | .095*** | .0026 | -.02 | .014 | -.0027 | .10*** | -.034* |
| *Note.* Point biserial correlation coefficient was calculated for dichotomous variables. Coding scheme as follows ^a^1 = Male, 2 = Female; ^b^1 = No, 2 = Yes; ^c^1 = Rural/Village, 2 = Urban/City; ^d^1 = Basic education (compulsory level), 2 = Secondary/high school, 3 = Professional school (incl. vocational school), 4 = University, 5 = Some post-graduate, 6 = Ph.D. | | | | | | | | | | | | | | |

| **Table S2**. Descriptive statistics (mean, SD) and frequencies (percent, n) for the control variables age, gender, ethnic status, and experienced discrimination by country and region. | | | | | | | | | | | | | | |
| --- | --- | --- | --- | --- | --- | --- | --- | --- | --- | --- | --- | --- | --- | --- |
|  | |  |  |  |  | |  |  |  | | ***Experienced Discrimination*** | | | |
|  | |  |  |  | ***Gender*** | | | | ***Status*** | | ***No*** | | ***Yes*** | |
| ***Country*** | | ***Region*** | ***n*** | ***Age***  ***M (SD)*** | ***Male***  ***% (n)*** | ***Female***  ***% (n)*** | ***Other***  ***% (n)*** | ***No answer***  ***% (n)*** | ***Majority***  ***% (n)*** | ***Minority***  ***% (n)*** | ***Majority***  ***% (n)*** | ***Minority***  ***% (n)*** | ***Majority***  ***% (n)*** | ***Minority***  ***% (n)*** |
| **Bosnia Herzegovina** | | | **923** | **22.2 (4.3)** | **49% (453)** | **51% (470)** | **0% (0)** | **0% (0)** | **53% (488)** | **47% (435)** | **50% (462)** | **40% (368)** | **3% (23)** | **7% (62)** |
|  | Unsko Sanski Canton | | 77 | 21.8 (4.3) | 48% (37) | 52% (40) | 0% (0) | 0% (0) | 87% (67) | 13% (10) | 86% (66) | 10% (8) | 1% (1) | 3% (2) |
|  | Posavski Canton | | 11 | 22.8 (4.1) | 45% (5) | 55% (6) | 0% (0) | 0% (0) | 36% (4) | 64% (7) | 27% (3) | 64% (7) | 9% (1) | 0% (0) |
|  | Tuzlanski Canton | | 121 | 22.1 (4.4) | 49% (59) | 51% (62) | 0% (0) | 0% (0) | 76% (92) | 24% (29) | 76% (91) | 18% (21) | 0% (0) | 7% (8) |
|  | Zenicko Dobojski Canton | | 101 | 21.6 (3.7) | 47% (47) | 53% (54) | 0% (0) | 0% (0) | 67% (68) | 33% (33) | 62% (63) | 27% (27) | 5% (5) | 6% (6) |
|  | Bosansko Podrinjski Canton | | 6 | 23.7 (4.7) | 50% (3) | 50% (3) | 0% (0) | 0% (0) | 100% (6) | 0% (0) | 100% (5) | 0% (0) | 0% (0) | 0% (0) |
|  | Srednjebosanski Canton | | 70 | 22.5 (4.3) | 49% (34) | 51% (36) | 0% (0) | 0% (0) | 87% (61) | 13% (9) | 86% (60) | 10% (7) | 1% (1) | 3% (2) |
| **Bosnia Herzegovina** | | |  |  |  |  |  |  |  |  |  |  |  |  |
|  | Hercegovacko Neretvanski Canton | | 57 | 22.2 (4) | 49% (28) | 51% (29) | 0% (0) | 0% (0) | 44% (25) | 56% (32) | 41% (23) | 43% (24) | 4% (2) | 12% (7) |
|  | Zapadnohercegovacki Canton | | 26 | 22 (4.8) | 46% (12) | 54% (14) | 0% (0) | 0% (0) | 0% (0) | 100% (26) | 0% (0) | 92% (24) | 0% (0) | 8% (2) |
|  | Canton Sarajevo | | 111 | 22.1 (4.1) | 55% (61) | 45% (50) | 0% (0) | 0% (0) | 41% (45) | 59% (66) | 40% (44) | 43% (48) | 1% (1) | 16% (18) |
|  | Canton 10 | | 21 | 22.1 (3.9) | 48% (10) | 52% (11) | 0% (0) | 0% (0) | 29% (6) | 71% (15) | 29% (6) | 71% (15) | 0% (0) | 0% (0) |
|  | Region Banja Luka | | 137 | 22.3 (4.3) | 50% (68) | 50% (69) | 0% (0) | 0% (0) | 40% (55) | 60% (82) | 34% (47) | 52% (71) | 6% (8) | 8% (11) |
|  | Region Doboj | | 49 | 22 (4.6) | 49% (24) | 51% (25) | 0% (0) | 0% (0) | 39% (19) | 61% (30) | 39% (19) | 53% (26) | 0% (0) | 8% (4) |
| **Bosnia Herzegovina** | | |  |  |  |  |  |  |  |  |  |  |  |  |
|  | Region Bijeljina | | 32 | 22.2 (5) | 47% (15) | 53% (17) | 0% (0) | 0% (0) | 34% (11) | 66% (21) | 31% (10) | 66% (21) | 3% (1) | 0% (0) |
|  | Region Zvornik | | 41 | 22.8 (4.6) | 49% (20) | 51% (21) | 0% (0) | 0% (0) | 59% (24) | 41% (17) | 50% (20) | 40% (16) | 8% (3) | 2% (1) |
|  | Region Pale | | 15 | 23.1 (4.7) | 47% (7) | 53% (8) | 0% (0) | 0% (0) | 0% (0) | 100% (15) | 0% (0) | 100% (14) | 0% (0) | 0% (0) |
|  | Region Foca | | 13 | 22.4 (4.9) | 46% (6) | 54% (7) | 0% (0) | 0% (0) | 0% (0) | 100% (13) | 0% (0) | 100% (12) | 0% (0) | 0% (0) |
|  | Region Trebinje | | 15 | 22.7 (5.3) | 47% (7) | 53% (8) | 0% (0) | 0% (0) | 0% (0) | 100% (15) | 0% (0) | 92% (12) | 0% (0) | 8% (1) |
|  | Region Distrikt Brcko | | 20 | 22.6 (4.2) | 50% (10) | 50% (10) | 0% (0) | 0% (0) | 25% (5) | 75% (15) | 25% (5) | 75% (15) | 0% (0) | 0% (0) |
| **Kosova** | | | **894** | **21.9 (4)** | **49% (442)** | **50% (449)** | **0% (2)** | **0% (1)** | **73% (650)** | **27% (244)** | **71% (619)** | **22% (194)** | **3% (22)** | **5% (41)** |
|  | Ferizaj | | 91 | 20.9 (4.4) | 40% (36) | 60% (55) | 0% (0) | 0% (0) | 86% (78) | 14% (13) | 87% (75) | 10% (9) | 2% (2) | 0% (0) |
|  | Gjakove | | 58 | 22.5 (4.6) | 43% (25) | 57% (33) | 0% (0) | 0% (0) | 79% (46) | 21% (12) | 79% (45) | 19% (11) | 0% (0) | 2% (1) |
|  | Gjilan | | 78 | 22.3 (3.8) | 56% (44) | 44% (34) | 0% (0) | 0% (0) | 77% (60) | 23% (18) | 75% (58) | 23% (18) | 1% (1) | 0% (0) |
|  | Mitrovice | | 165 | 22.4 (3.9) | 55% (90) | 45% (75) | 0% (0) | 0% (0) | 49% (81) | 51% (84) | 48% (77) | 43% (69) | 1% (2) | 9% (14) |
|  | Peje | | 96 | 20.8 (3.3) | 52% (50) | 47% (45) | 1% (1) | 0% (0) | 69% (66) | 31% (30) | 64% (61) | 28% (27) | 4% (4) | 3% (3) |
|  | Prishtine | | 197 | 22.7 (3.9) | 50% (99) | 49% (96) | 1% (1) | 1% (1) | 91% (179) | 9% (18) | 87% (169) | 7% (13) | 4% (8) | 3% (5) |
| **Kosova** | | |  |  |  |  |  |  |  |  |  |  |  |  |
|  | Prizren | | 209 | 21.5 (4.1) | 47% (98) | 53% (111) | 0% (0) | 0% (0) | 67% (140) | 33% (69) | 66% (134) | 23% (47) | 2% (5) | 9% (18) |
| **Montenegro** | | | **896** | **21.8 (4.3)** | **48% (434)** | **51% (454)** | **1% (6)** | **0% (2)** | **43% (382)** | **57% (514)** | **40% (355)** | **51% (456)** | **3% (26)** | **6% (51)** |
|  | Central Region | | 456 | 21.5 (4.3) | 49% (224) | 50% (227) | 1% (5) | 0% (0) | 49% (222) | 51% (234) | 46% (211) | 46% (210) | 2% (10) | 5% (24) |
|  | Coastal Region | | 202 | 21.6 (4.2) | 50% (101) | 49% (98) | 0% (1) | 1% (2) | 42% (84) | 58% (118) | 40% (80) | 56% (111) | 2% (4) | 2% (4) |
|  | Northern Region | | 238 | 22.7 (4.2) | 46% (109) | 54% (129) | 0% (0) | 0% (0) | 32% (76) | 68% (162) | 27% (64) | 58% (135) | 5% (12) | 10% (23) |
| **North Macedonia** | | | **896** | **22.6 (4.2)** | **50% (446)** | **50% (447)** | **0% (1)** | **0% (2)** | **66% (587)** | **34% (309)** | **64% (564)** | **29% (260)** | **2% (21)** | **5% (41)** |
|  | Vardar Region | | 90 | 21.2 (4) | 46% (41) | 54% (49) | 0% (0) | 0% (0) | 68% (61) | 32% (29) | 68% (61) | 23% (21) | 0% (0) | 9% (8) |
| **North Macedonia** | | |  |  |  |  |  |  |  |  |  |  |  |  |
|  | Eastern Region | | 74 | 22.9 (4.2) | 47% (35) | 53% (39) | 0% (0) | 0% (0) | 100% (74) | 0% (0) | 100% (74) | 0% (0) | 0% (0) | 0% (0) |
|  | South Western Region | | 82 | 22.2 (4.4) | 55% (45) | 45% (37) | 0% (0) | 0% (0) | 60% (49) | 40% (33) | 55% (44) | 30% (24) | 5% (4) | 10% (8) |
|  | South Eastern Region | | 80 | 22.4 (4.3) | 51% (41) | 49% (39) | 0% (0) | 0% (0) | 88% (70) | 12% (10) | 86% (69) | 12% (10) | 1% (1) | 0% (0) |
|  | Pelagonija Region | | 126 | 22 (4.2) | 52% (66) | 48% (60) | 0% (0) | 0% (0) | 70% (88) | 30% (38) | 69% (85) | 27% (34) | 2% (2) | 2% (3) |
|  | Polog Region | | 117 | 22.9 (4.3) | 46% (54) | 54% (63) | 0% (0) | 0% (0) | 29% (34) | 71% (83) | 29% (34) | 65% (75) | 0% (0) | 6% (7) |
|  | North Eastern Region | | 75 | 23.3 (3.8) | 52% (39) | 48% (36) | 0% (0) | 0% (0) | 59% (44) | 41% (31) | 61% (44) | 33% (24) | 0% (0) | 6% (4) |
|  | Skopje Region | | 252 | 23.2 (4) | 50% (125) | 49% (124) | 0% (1) | 1% (2) | 66% (167) | 34% (85) | 61% (153) | 29% (72) | 6% (14) | 4% (11) |
| **Serbia** | | | **900** | **22.3 (4.2)** | **50% (451)** | **50% (449)** | **0% (0)** | **0% (0)** | **72% (651)** | **28% (249)** | **71% (625)** | **24% (210)** | **2% (14)** | **4% (37)** |
|  | Beograd | | 169 | 22.3 (4.2) | 51% (86) | 49% (83) | 0% (0) | 0% (0) | 97% (164) | 3% (5) | 96% (160) | 2% (3) | 1% (2) | 1% (2) |
|  | Vojvodina | | 196 | 22.4 (4.2) | 49% (97) | 51% (99) | 0% (0) | 0% (0) | 90% (176) | 10% (20) | 89% (174) | 9% (17) | 1% (2) | 2% (3) |
|  | Central Western Serbia | | 322 | 22.2 (4.3) | 50% (162) | 50% (160) | 0% (0) | 0% (0) | 54% (175) | 46% (147) | 50% (158) | 44% (137) | 3% (9) | 3% (9) |
|  | South Eastern Serbia | | 213 | 22.2 (4.2) | 50% (106) | 50% (107) | 0% (0) | 0% (0) | 64% (136) | 36% (77) | 63% (133) | 25% (53) | 0% (1) | 11% (23) |

| **Table S3.** Frequencies (percent, n) for the control variables urbanity, mobility, and engagement by country and region. | | | | | | | | | |
| --- | --- | --- | --- | --- | --- | --- | --- | --- | --- |
|  | |  |  | ***Urbanity*** | | ***Mobility*** | | ***Engagement*** | |
| ***Country*** | | ***Region*** | ***n*** | ***Urban/City % (n)*** | ***Rural/Village % (n)*** | ***Yes***  ***% (n)*** | ***No***  ***% (n)*** | ***Yes***  ***% (n)*** | ***No***  ***% (n)*** |
| **Bosnia Herzegovina** | |  | **923** | **47% (437)** | **53% (486)** | **40% (371)** | **60% (552)** | **26% (240)** | **74% (683)** |
|  | Unsko Sanski Canton | | 77 | 39% (30) | 61% (47) | 51% (39) | 49% (38) | 34% (26) | 66% (51) |
|  | Posavski Canton | | 11 | 45% (5) | 55% (6) | 27% (3) | 73% (8) | 0% (0) | 100% (11) |
|  | Tuzlanski Canton | | 121 | 37% (45) | 63% (76) | 38% (46) | 62% (75) | 17% (21) | 83% (100) |
|  | Zenicko Dobojski Canton | | 101 | 38% (38) | 62% (63) | 34% (34) | 66% (67) | 49% (49) | 51% (52) |
|  | Bosansko Podrinjski Canton | | 6 | 50% (3) | 50% (3) | 67% (4) | 33% (2) | 33% (2) | 67% (4) |
|  | Srednjebosanski Canton | | 70 | 33% (23) | 67% (47) | 46% (32) | 54% (38) | 27% (19) | 73% (51) |
|  | Hercegovacko Neretvanski Canton | | 57 | 47% (27) | 53% (30) | 35% (20) | 65% (37) | 30% (17) | 70% (40) |
|  | Zapadnohercegovacki Canton | | 26 | 23% (6) | 77% (20) | 46% (12) | 54% (14) | 12% (3) | 88% (23) |
|  | Canton Sarajevo | | 111 | 92% (102) | 8% (9) | 56% (62) | 44% (49) | 23% (26) | 77% (85) |
| **Bosnia Herzegovina** | |  |  |  |  |  |  |  |  |
|  | Canton 10 | | 21 | 29% (6) | 71% (15) | 38% (8) | 62% (13) | 19% (4) | 81% (17) |
|  | Region Banja Luka | | 137 | 49% (67) | 51% (70) | 46% (63) | 54% (74) | 35% (48) | 65% (89) |
|  | Region Doboj | | 49 | 35% (17) | 65% (32) | 27% (13) | 73% (36) | 24% (12) | 76% (37) |
|  | Region Bijeljina | | 32 | 38% (12) | 62% (20) | 12% (4) | 88% (28) | 19% (6) | 81% (26) |
|  | Region Zvornik | | 41 | 41% (17) | 59% (24) | 20% (8) | 80% (33) | 7% (3) | 93% (38) |
|  | Region Pale | | 15 | 67% (10) | 33% (5) | 7% (1) | 93% (14) | 0% (0) | 100% (15) |
|  | Region Foca | | 13 | 54% (7) | 46% (6) | 8% (1) | 92% (12) | 8% (1) | 92% (12) |
|  | Region Trebinje | | 15 | 73% (11) | 27% (4) | 7% (1) | 93% (14) | 20% (3) | 80% (12) |
|  | Region Distrikt Brcko | | 20 | 55% (11) | 45% (9) | 100% (20) | 0% (0) | 0% (0) | 100% (20) |
| **Kosova** | |  | **894** | **46% (412)** | **54% (482)** | **18% (163)** | **82% (731)** | **16% (147)** | **84% (747)** |
|  | Ferizaj | | 91 | 43% (39) | 57% (52) | 10% (9) | 90% (82) | 15% (14) | 85% (77) |
| **Kosova** | |  |  |  |  |  |  |  |  |
|  | Gjakove | | 58 | 53% (31) | 47% (27) | 12% (7) | 88% (51) | 24% (14) | 76% (44) |
|  | Gjilan | | 78 | 31% (24) | 69% (54) | 14% (11) | 86% (67) | 12% (9) | 88% (69) |
|  | Mitrovice | | 165 | 68% (113) | 32% (52) | 18% (29) | 82% (136) | 19% (32) | 81% (133) |
|  | Peje | | 96 | 34% (33) | 66% (63) | 18% (17) | 82% (79) | 25% (24) | 75% (72) |
|  | Prishtine | | 197 | 45% (89) | 55% (108) | 20% (39) | 80% (158) | 12% (23) | 88% (174) |
|  | Prizren | | 209 | 40% (83) | 60% (126) | 24% (51) | 76% (158) | 15% (31) | 85% (178) |
| **Montenegro** | |  | **896** | **67% (600)** | **33% (296)** | **39% (353)** | **61% (543)** | **33% (293)** | **67% (603)** |
|  | Central Region | | 456 | 74% (339) | 26% (117) | 32% (147) | 68% (309) | 30% (137) | 70% (319) |
|  | Coastal Region | | 202 | 64% (129) | 36% (73) | 51% (104) | 49% (98) | 37% (75) | 63% (127) |
|  | Northern Region | | 238 | 55% (132) | 45% (106) | 43% (102) | 57% (136) | 34% (81) | 66% (157) |
| **North Macedonia** | |  | **896** | **62% (560)** | **38% (336)** | **45% (407)** | **55% (489)** | **17% (156)** | **83% (740)** |
|  | Vardar Region | | 90 | 70% (63) | 30% (27) | 17% (15) | 83% (75) | 9% (8) | 91% (82) |
|  | Eastern Region | | 74 | 66% (49) | 34% (25) | 51% (38) | 49% (36) | 5% (4) | 95% (70) |
|  | South Western Region | | 82 | 57% (47) | 43% (35) | 60% (49) | 40% (33) | 26% (21) | 74% (61) |
|  | South Eastern Region | | 80 | 49% (39) | 51% (41) | 54% (43) | 46% (37) | 16% (13) | 84% (67) |
|  | Pelagonija Region | | 126 | 78% (98) | 22% (28) | 36% (45) | 64% (81) | 10% (13) | 90% (113) |
|  | Polog Region | | 117 | 35% (41) | 65% (76) | 44% (52) | 56% (65) | 24% (28) | 76% (89) |
|  | North Eastern Region | | 75 | 63% (47) | 37% (28) | 49% (37) | 51% (38) | 20% (15) | 80% (60) |
|  | Skopje Region | | 252 | 70% (176) | 30% (76) | 51% (128) | 49% (124) | 21% (54) | 79% (198) |
| **Serbia** | |  | **900** | **59% (528)** | **41% (372)** | **38% (340)** | **62% (560)** | **14% (124)** | **86% (776)** |
|  | Beograd | | 169 | 81% (137) | 19% (32) | 55% (93) | 45% (76) | 24% (41) | 76% (128) |
|  | Vojvodina | | 196 | 59% (116) | 41% (80) | 42% (83) | 58% (113) | 8% (16) | 92% (180) |
| **Serbia** | |  |  |  |  |  |  |  |  |
|  | Central Western Serbia | | 322 | 53% (171) | 47% (151) | 30% (98) | 70% (224) | 11% (36) | 89% (286) |
|  | South Eastern Serbia | | 213 | 49% (104) | 51% (109) | 31% (66) | 69% (147) | 15% (31) | 85% (182) |

| **Table S4.** Frequencies (percent, n) for the control variable education by country and region. | | | | | | | | | |
| --- | --- | --- | --- | --- | --- | --- | --- | --- | --- |
| ***Country*** | | ***Region*** | ***n*** | ***Basic Education/***  ***Compulsory Level % (n)*** | ***Secondary High School***  ***% (n)*** | ***Professional School***  ***% (n)*** | ***University***  ***% (n)*** | ***Post Graduate***  ***% (n)*** | ***No Answer***  ***% (n)*** |
| **Bosnia Herzegovina** | |  | **923** | **14% (132)** | **57% (530)** | **7% (64)** | **19% (175)** | **2% (20)** | **0% (2)** |
|  | Unsko Sanski Canton | | 77 | 25% (19) | 51% (39) | 6% (5) | 16% (12) | 3% (2) | 0% (0) |
|  | Posavski Canton | | 11 | 0% (0) | 91% (10) | 0% (0) | 9% (1) | 0% (0) | 0% (0) |
|  | Tuzlanski Canton | | 121 | 18% (22) | 60% (72) | 2% (3) | 19% (23) | 1% (1) | 0% (0) |
|  | Zenicko Dobojski Canton | | 101 | 1% (1) | 83% (84) | 1% (1) | 12% (12) | 3% (3) | 0% (0) |
|  | Bosansko Podrinjski Canton | | 6 | 17% (1) | 50% (3) | 0% (0) | 33% (2) | 0% (0) | 0% (0) |
|  | Srednjebosanski Canton | | 70 | 11% (8) | 43% (30) | 9% (6) | 27% (19) | 10% (7) | 0% (0) |
|  | Hercegovacko Neretvanski Canton | | 57 | 11% (6) | 49% (28) | 12% (7) | 25% (14) | 4% (2) | 0% (0) |
|  | Zapadnohercegovacki Canton | | 26 | 15% (4) | 58% (15) | 12% (3) | 8% (2) | 8% (2) | 0% (0) |
|  | Canton Sarajevo | | 111 | 16% (18) | 54% (60) | 4% (4) | 25% (28) | 1% (1) | 0% (0) |
| **Bosnia Herzegovina** | |  |  |  |  |  |  |  |  |
|  | Canton 10 | | 21 | 5% (1) | 57% (12) | 10% (2) | 29% (6) | 0% (0) | 0% (0) |
|  | Region Banja Luka | | 137 | 18% (25) | 53% (72) | 7% (9) | 22% (30) | 0% (0) | 1% (1) |
|  | Region Doboj | | 49 | 27% (13) | 61% (30) | 2% (1) | 10% (5) | 0% (0) | 0% (0) |
|  | Region Bijeljina | | 32 | 25% (8) | 59% (19) | 3% (1) | 6% (2) | 3% (1) | 3% (1) |
|  | Region Zvornik | | 41 | 5% (2) | 71% (29) | 10% (4) | 15% (6) | 0% (0) | 0% (0) |
|  | Region Pale | | 15 | 0% (0) | 40% (6) | 47% (7) | 13% (2) | 0% (0) | 0% (0) |
|  | Region Foca | | 13 | 15% (2) | 15% (2) | 54% (7) | 15% (2) | 0% (0) | 0% (0) |
|  | Region Trebinje | | 15 | 13% (2) | 40% (6) | 27% (4) | 13% (2) | 7% (1) | 0% (0) |
|  | Region Distrikt Brcko | | 20 | 0% (0) | 65% (13) | 0% (0) | 35% (7) | 0% (0) | 0% (0) |
| **Kosova** | |  | **894** | **23% (204)** | **41% (369)** | **11% (96)** | **22% (197)** | **3% (28)** | **0% (0)** |
|  | Ferizaj | | 91 | 31% (28) | 51% (46) | 8% (7) | 9% (8) | 2% (2) | 0% (0) |
|  | Gjakove | | 58 | 21% (12) | 52% (30) | 3% (2) | 22% (13) | 2% (1) | 0% (0) |
| **Kosova** | |  |  |  |  |  |  |  |  |
|  | Gjilan | | 78 | 18% (14) | 46% (36) | 12% (9) | 22% (17) | 3% (2) | 0% (0) |
|  | Mitrovice | | 165 | 20% (33) | 34% (56) | 21% (34) | 22% (37) | 3% (5) | 0% (0) |
|  | Peje | | 96 | 36% (35) | 43% (41) | 1% (1) | 17% (16) | 3% (3) | 0% (0) |
|  | Prishtine | | 197 | 13% (25) | 31% (62) | 13% (26) | 38% (74) | 5% (10) | 0% (0) |
|  | Prizren | | 209 | 27% (57) | 47% (98) | 8% (17) | 15% (32) | 2% (5) | 0% (0) |
| **Montenegro** | |  | **896** | **25% (228)** | **27% (239)** | **24% (211)** | **16% (144)** | **6% (54)** | **2% (20)** |
|  | Central Region | | 456 | 31% (141) | 26% (119) | 25% (112) | 12% (57) | 5% (24) | 1% (3) |
|  | Coastal Region | | 202 | 19% (38) | 34% (69) | 19% (39) | 20% (41) | 3% (6) | 4% (9) |
|  | Northern Region | | 238 | 21% (49) | 21% (51) | 25% (60) | 19% (46) | 10% (24) | 3% (8) |
| **North Macedonia** | |  | **896** | **22% (201)** | **25% (223)** | **32% (284)** | **18% (164)** | **3% (24)** | **0% (0)** |
|  | Vardar Region | | 90 | 36% (32) | 22% (20) | 30% (27) | 11% (10) | 1% (1) | 0% (0) |
|  | Eastern Region | | 74 | 16% (12) | 27% (20) | 38% (28) | 16% (12) | 3% (2) | 0% (0) |
| **North Macedonia** | |  |  |  |  |  |  |  |  |
|  | South Western Region | | 82 | 24% (20) | 18% (15) | 32% (26) | 22% (18) | 4% (3) | 0% (0) |
|  | South Eastern Region | | 80 | 22% (18) | 32% (26) | 30% (24) | 15% (12) | 0% (0) | 0% (0) |
|  | Pelagonija Region | | 126 | 33% (42) | 18% (23) | 37% (47) | 10% (13) | 1% (1) | 0% (0) |
|  | Polog Region | | 117 | 18% (21) | 21% (24) | 32% (38) | 24% (28) | 5% (6) | 0% (0) |
|  | North Eastern Region | | 75 | 24% (18) | 33% (25) | 31% (23) | 7% (5) | 5% (4) | 0% (0) |
|  | Skopje Region | | 252 | 15% (38) | 28% (70) | 28% (71) | 26% (66) | 3% (7) | 0% (0) |
| **Serbia** | |  | **900** | **28% (255)** | **9% (84)** | **51% (459)** | **10% (89)** | **1% (12)** | **0% (1)** |
|  | Beograd | | 169 | 23% (39) | 20% (34) | 40% (68) | 13% (22) | 4% (6) | 0% (0) |
|  | Vojvodina | | 196 | 25% (49) | 7% (14) | 56% (109) | 12% (24) | 0% (0) | 0% (0) |
|  | Central Western Serbia | | 322 | 31% (101) | 8% (26) | 51% (164) | 8% (25) | 2% (5) | 0% (1) |
|  | South Eastern Serbia | | 213 | 31% (66) | 5% (10) | 55% (118) | 8% (18) | 0% (1) | 0% (0) |

| **Table S5***.* Frequencies (percent, n) for the control variable ethnicity by country and region. | | | | | | | | | | | | | | | |
| --- | --- | --- | --- | --- | --- | --- | --- | --- | --- | --- | --- | --- | --- | --- | --- |
| ***Country*** | | ***Region*** | | ***n*** | ***Bosniak***  ***% (n)*** | ***Croat***  ***% (n)*** | ***Roma***  ***% (n)*** | ***Serb***  ***% (n)*** | ***Monte-negrin***  ***% (n)*** | ***Albanian % (n)*** | ***Maced-onian % (n)*** | ***Turk***  ***% (n)*** | ***Hunga-rian***  ***% (n)*** | ***Other***  ***% (n)*** | ***Ethnic Fract.*** |
| **Bosnia Herzegovina** | | | | **923** | **53% (485)** | **13% (118)** | **4% (35)** | **30% (279)** | **0% (2)** | **0% (1)** | **0% (0)** | **0% (0)** | **0% (0)** | **0% (3)** | **0.454** |
|  | Unsko Sanski Canton | | | 77 | 87% (67) | 3% (2) | 1% (1) | 9% (7) | 0% (0) | 0% (0) | 0% (0) | 0% (0) | 0% (0) | 0% (0) | 0.23 |
|  | Posavski Canton | | | 11 | 36% (4) | 64% (7) | 0% (0) | 0% (0) | 0% (0) | 0% (0) | 0% (0) | 0% (0) | 0% (0) | 0% (0) | 0.46 |
|  | Tuzlanski Canton | | | 121 | 76% (92) | 1% (1) | 5% (6) | 18% (22) | 0% (0) | 0% (0) | 0% (0) | 0% (0) | 0% (0) | 0% (0) | 0.39 |
|  | Zenicko Dobojski Canton | | | 101 | 66% (67) | 15% (15) | 3% (3) | 15% (15) | 0% (0) | 0% (0) | 0% (0) | 0% (0) | 0% (0) | 1% (1) | 0.51 |
|  | Bosansko Podrinjski Canton | | | 6 | 100% (6) | 0% (0) | 0% (0) | 0% (0) | 0% (0) | 0% (0) | 0% (0) | 0% (0) | 0% (0) | 0% (0) | 0.00 |
|  | Srednjebosanski Canton | | | 70 | 87% (61) | 9% (6) | 0% (0) | 4% (3) | 0% (0) | 0% (0) | 0% (0) | 0% (0) | 0% (0) | 0% (0) | 0.23 |
|  | Hercegovacko Neretvanski Canton | | | 57 | 44% (25) | 42% (24) | 2% (1) | 11% (6) | 2% (1) | 0% (0) | 0% (0) | 0% (0) | 0% (0) | 0% (0) | 0.63 |
| **Bosnia Herzegovina** | | | |  |  |  |  |  |  |  |  |  |  |  |  |
|  | Zapadnohercegovacki Canton | | | 26 | 0% (0) | 100% (26) | 0% (0) | 0% (0) | 0% (0) | 0% (0) | 0% (0) | 0% (0) | 0% (0) | 0% (0) | 0.00 |
|  | Canton Sarajevo | | | 111 | 41% (45) | 9% (10) | 6% (7) | 43% (48) | 1% (1) | 0% (0) | 0% (0) | 0% (0) | 0% (0) | 0% (0) | 0.64 |
|  | Canton 10 | | | 21 | 29% (6) | 57% (12) | 0% (0) | 14% (3) | 0% (0) | 0% (0) | 0% (0) | 0% (0) | 0% (0) | 0% (0) | 0.57 |
|  | Region Banja Luka | | | 137 | 39% (53) | 8% (11) | 9% (12) | 42% (58) | 0% (0) | 1% (1) | 0% (0) | 0% (0) | 0% (0) | 1% (2) | 0.65 |
|  | Region Doboj | | | 49 | 39% (19) | 8% (4) | 4% (2) | 49% (24) | 0% (0) | 0% (0) | 0% (0) | 0% (0) | 0% (0) | 0% (0) | 0.60 |
|  | Region Bijeljina | | | 32 | 34% (11) | 0% (0) | 6% (2) | 59% (19) | 0% (0) | 0% (0) | 0% (0) | 0% (0) | 0% (0) | 0% (0) | 0.53 |
|  | Region Zvornik | | | 41 | 59% (24) | 0% (0) | 2% (1) | 39% (16) | 0% (0) | 0% (0) | 0% (0) | 0% (0) | 0% (0) | 0% (0) | 0.48 |
|  | Region Pale | | | 15 | 0% (0) | 0% (0) | 0% (0) | 100% (15) | 0% (0) | 0% (0) | 0% (0) | 0% (0) | 0% (0) | 0% (0) | 0.00 |
| **Bosnia Herzegovina** | | | |  |  |  |  |  |  |  |  |  |  |  |  |
|  | Region Foca | | | 13 | 0% (0) | 0% (0) | 0% (0) | 100% (13) | 0% (0) | 0% (0) | 0% (0) | 0% (0) | 0% (0) | 0% (0) | 0.00 |
|  | Region Trebinje | | | 15 | 0% (0) | 0% (0) | 0% (0) | 100% (15) | 0% (0) | 0% (0) | 0% (0) | 0% (0) | 0% (0) | 0% (0) | 0.00 |
|  | Region Distrikt Brcko | | | 20 | 25% (5) | 0% (0) | 0% (0) | 75% (15) | 0% (0) | 0% (0) | 0% (0) | 0% (0) | 0% (0) | 0% (0) | 0.38 |
| **Kosova** | | |  | **894** | **8% (70)** | **0% (0)** | **8% (73)** | **11% (100)** | **0% (1)** | **73% (650)** | **0% (0)** | **0% (0)** | **0% (0)** | **0% (0)** | **0.385** |
|  | Ferizaj | | | 91 | 0% (0) | 0% (0) | 13% (12) | 1% (1) | 0% (0) | 86% (78) | 0% (0) | 0% (0) | 0% (0) | 0% (0) | 0.25 |
|  | Gjakove | | | 58 | 0% (0) | 0% (0) | 19% (11) | 2% (1) | 0% (0) | 79% (46) | 0% (0) | 0% (0) | 0% (0) | 0% (0) | 0.32 |
|  | Gjilan | | | 78 | 0% (0) | 0% (0) | 0% (0) | 23% (18) | 0% (0) | 77% (60) | 0% (0) | 0% (0) | 0% (0) | 0% (0) | 0.35 |
|  | Mitrovice | | | 165 | 1% (1) | 0% (0) | 10% (16) | 41% (67) | 0% (0) | 49% (81) | 0% (0) | 0% (0) | 0% (0) | 0% (0) | 0.58 |
| **Kosova** | | |  |  |  |  |  |  |  |  |  |  |  |  |  |
|  | Peje | | | 96 | 21% (20) | 0% (0) | 10% (10) | 0% (0) | 0% (0) | 69% (66) | 0% (0) | 0% (0) | 0% (0) | 0% (0) | 0.47 |
|  | Prishtine | | | 197 | 0% (0) | 0% (0) | 3% (5) | 7% (13) | 0% (0) | 91% (179) | 0% (0) | 0% (0) | 0% (0) | 0% (0) | 0.17 |
|  | Prizren | | | 209 | 23% (49) | 0% (0) | 9% (19) | 0% (0) | 0% (1) | 67% (140) | 0% (0) | 0% (0) | 0% (0) | 0% (0) | 0.48 |
| **Montenegro** | | |  | **896** | **9% (83)** | **0% (3)** | **12% (108)** | **20% (179)** | **43% (381)** | **15% (138)** | **0% (0)** | **0% (0)** | **0% (0)** | **0% (4)** | **0.688** |
|  | Central Region | | | 456 | 1% (6) | 0% (0) | 21% (96) | 15% (70) | 49% (223) | 12% (57) | 0% (0) | 0% (0) | 0% (0) | 1% (4) | 0.68 |
|  | Coastal Region | | | 202 | 5% (11) | 1% (2) | 0% (0) | 20% (40) | 42% (84) | 32% (65) | 0% (0) | 0% (0) | 0% (0) | 0% (0) | 0.68 |
|  | Northern Region | | | 238 | 28% (66) | 0% (1) | 5% (12) | 29% (69) | 31% (74) | 7% (16) | 0% (0) | 0% (0) | 0% (0) | 0% (0) | 0.72 |
| **North Macedonia** | | | | **896** | **0% (4)** | **0% (0)** | **13% (113)** | **1% (5)** | **0% (1)** | **18% (163)** | **67% (596)** | **2% (14)** | **0% (0)** | **0% (0)** | **0.419** |
|  | Vardar Region | | | 90 | 0% (0) | 0% (0) | 32% (29) | 0% (0) | 0% (0) | 0% (0) | 68% (61) | 0% (0) | 0% (0) | 0% (0) | 0.44 |
|  | Eastern Region | | | 74 | 3% (2) | 0% (0) | 0% (0) | 0% (0) | 0% (0) | 0% (0) | 97% (72) | 0% (0) | 0% (0) | 0% (0) | -0.00 |
|  | South Western Region | | | 82 | 0% (0) | 0% (0) | 1% (1) | 0% (0) | 0% (0) | 33% (27) | 60% (49) | 6% (5) | 0% (0) | 0% (0) | 0.54 |
|  | South Eastern Region | | | 80 | 0% (0) | 0% (0) | 10% (8) | 0% (0) | 0% (0) | 0% (0) | 88% (70) | 2% (2) | 0% (0) | 0% (0) | 0.21 |
|  | Pelagonija Region | | | 126 | 0% (0) | 0% (0) | 26% (33) | 0% (0) | 1% (1) | 0% (0) | 71% (90) | 2% (2) | 0% (0) | 0% (0) | 0.42 |
|  | Polog Region | | | 117 | 0% (0) | 0% (0) | 9% (10) | 0% (0) | 0% (0) | 62% (73) | 29% (34) | 0% (0) | 0% (0) | 0% (0) | 0.51 |
|  | North Eastern Region | | | 75 | 0% (0) | 0% (0) | 13% (10) | 4% (3) | 0% (0) | 24% (18) | 59% (44) | 0% (0) | 0% (0) | 0% (0) | 0.58 |
|  | Skopje Region | | | 252 | 1% (2) | 0% (0) | 9% (22) | 1% (2) | 0% (0) | 18% (45) | 70% (176) | 2% (5) | 0% (0) | 0% (0) | 0.47 |
| **Serbia** | | |  | **900** | **12% (107)** | **1% (5)** | **13% (118)** | **71% (642)** | **0% (4)** | **0% (2)** | **1% (5)** | **0% (0)** | **2% (14)** | **0% (3)** | **0.366** |
|  | Beograd | | | 169 | 1% (1) | 1% (1) | 1% (2) | 95% (161) | 1% (2) | 0% (0) | 1% (1) | 0% (0) | 0% (0) | 1% (1) | 0.05 |
|  | Vojvodina | | | 196 | 0% (0) | 2% (3) | 1% (2) | 89% (175) | 0% (0) | 0% (0) | 1% (1) | 0% (0) | 7% (14) | 1% (1) | 0.19 |
|  | Central Western Serbia | | | 322 | 33% (106) | 0% (1) | 12% (38) | 54% (173) | 1% (2) | 0% (0) | 1% (2) | 0% (0) | 0% (0) | 0% (0) | 0.58 |
|  | South Eastern Serbia | | | 213 | 0% (0) | 0% (0) | 36% (76) | 62% (133) | 0% (0) | 1% (2) | 0% (1) | 0% (0) | 0% (0) | 0% (1) | 0.46 |

| **Table S6***.* Means and standard deviations for Contact, Approach, Social Distance, Forgiveness, and Social Trust by country and by region. | | | | | | | | |
| --- | --- | --- | --- | --- | --- | --- | --- | --- |
| ***Country*** | | ***Region*** | ***n*** | ***Contact***  ***M (SD)*** | ***Appoach***  ***M (SD)*** | ***Social Distance***  ***M (SD)*** | ***Forgiveness***  ***M (SD)*** | ***Social Trust***  ***M (SD)*** |
| **Bosnia Herzegovina** | | | **923** | **2.83 (0.87)** | **3.51 (0.85)** | **2.67 (1.15)** | **3.42 (1.02)** | **3.39 (0.98)** |
|  | Unsko Sanski Canton | | 77 | 2.84 (0.73) | 3.56 (0.75) | 2.96 (1.1) | 2.94 (0.92) | 2.80 (0.78) |
|  | Posavski Canton | | 11 | 2.82 (0.6) | 3.18 (0.71) | 3.2 (0.66) | 3.45 (0.82) | 3.00 (0.89) |
|  | Tuzlanski Canton | | 121 | 2.98 (0.9) | 3.67 (0.8) | 2.51 (1.1) | 3.79 (0.76) | 3.67 (0.98) |
|  | Zenicko Dobojski Canton | | 101 | 2.32 (0.84) | 3.75 (0.78) | 2.41 (1.17) | 3.76 (1.04) | 3.48 (0.97) |
|  | Bosansko Podrinjski Canton | | 6 | 2.33 (0.68) | 3.3 (0.54) | 2.5 (1.44) | 2.4 (1.14) | 2.83 (1.17) |
|  | Srednjebosanski Canton | | 70 | 2.25 (0.79) | 3.67 (0.68) | 2.26 (0.94) | 3.41 (0.99) | 3.53 (0.99) |
|  | Hercegovacko Neretvanski Canton | | 57 | 3.03 (0.73) | 3.4 (0.54) | 2.88 (0.88) | 2.94 (0.87) | 2.96 (0.65) |
|  | Zapadnohercegovacki Canton | | 26 | 2.17 (0.6) | 3.03 (0.59) | 4.04 (0.78) | 2.84 (1.07) | 2.52 (0.95) |
|  | Canton Sarajevo | | 111 | 3.1 (0.78) | 3.7 (0.85) | 2.8 (1.21) | 3.18 (1.04) | 3.50 (0.91) |
|  | Canton 10 | | 21 | 3.64 (0.45) | 4.18 (0.62) | 1.73 (0.88) | 3.9 (0.7) | 4.10 (0.83) |
|  | Region Banja Luka | | 137 | 3.05 (0.77) | 3.33 (0.94) | 2.59 (1.17) | 3.81 (1) | 3.84 (0.82) |
|  | Region Doboj | | 49 | 2.92 (0.91) | 3.11 (0.91) | 3.06 (1.08) | 3.31 (1) | 3.24 (0.99) |
|  | Region Bijeljina | | 32 | 2.94 (0.72) | 3.01 (0.77) | 2.66 (1.41) | 3.69 (0.86) | 2.97 (1.12) |
| **Bosnia Herzegovina** | | |  |  |  |  |  |  |
|  | Region Zvornik | | 41 | 2.98 (0.7) | 3.27 (0.88) | 2.79 (1.07) | 3.00 (0.92) | 3.46 (0.79) |
|  | Region Pale | | 15 | 2.37 (0.55) | 3.02 (0.31) | 2.97 (0.27) | 2.93 (1.53) | 2.93 (1.10) |
|  | Region Foca | | 13 | 2.27 (0.53) | 3.21 (0.61) | 2.83 (0.52) | 3.23 (1.09) | 3.38 (1.19) |
|  | Region Trebinje | | 15 | 1.60 (0.51) | 2.90 (1.25) | 3.23 (0.67) | 2.60 (0.91) | 3.07 (1.03) |
|  | Region Distrikt Brcko | | 20 | 4.00 (0.00) | 4.58 (0.60) | 1.57 (1.14) | 2.94 (0.44) | 2.47 (0.70) |
| **Kosovo** | | | **894** | **1.90 (0.91)** | **2.38 (1.03)** | **3.48 (1.29)** | **2.32 (1.19)** | **2.62 (1.04)** |
|  | Ferizaj | | 91 | 1.66 (0.79) | 2.24 (1.00) | 3.20 (1.31) | 2.55 (1.13) | 2.64 (1.06) |
|  | Gjakove | | 58 | 1.83 (0.80) | 2.21 (0.96) | 4.00 (1.30) | 2.34 (0.71) | 2.54 (0.82) |
|  | Gjilan | | 78 | 1.71 (0.67) | 1.99 (0.71) | 3.94 (1.01) | 2.29 (1.15) | 2.27 (0.9) |
|  | Mitrovice | | 165 | 1.96 (0.85) | 2.18 (1.02) | 3.50 (1.07) | 2.38 (1.36) | 2.34 (1.05) |
|  | Peje | | 96 | 2.36 (0.86) | 2.55 (1.06) | 3.14 (1.31) | 2.78 (1.16) | 2.85 (1.07) |
|  | Prishtine | | 197 | 1.64 (0.86) | 2.32 (0.96) | 3.62 (1.34) | 2.09 (1.18) | 2.77 (1.10) |
|  | Prizren | | 209 | 2.07 (1.03) | 2.78 (1.11) | 3.30 (1.35) | 2.19 (1.15) | 2.72 (0.99) |
| **Montenegro** | | | **896** | **2.82 (0.87)** | **3.39 (0.89)** | **2.99 (1.15)** | **3.49 (1.00)** | **3.40 (1.01)** |
|  | Central Region | | 456 | 2.74 (0.89) | 3.43 (0.85) | 2.87 (1.12) | 3.47 (1.01) | 3.49 (0.94) |
|  | Coastal Region | | 202 | 3.05 (0.84) | 3.31 (1.02) | 2.95 (1.33) | 3.43 (1.04) | 3.02 (1.19) |
|  | Northern Region | | 238 | 2.79 (0.83) | 3.38 (0.85) | 3.25 (0.99) | 3.58 (0.94) | 3.55 (0.87) |
| **North Macedonia** | | | **896** | **2.62 (0.93)** | **2.94 (1.02)** | **3.42 (1.18)** | **3.17 (0.92)** | **3.16 (1.06)** |
|  | Vardar Region | | 90 | 2.72 (0.93) | 3.12 (0.93) | 3.84 (0.82) | 3.32 (0.81) | 3.69 (0.83) |
|  | Eastern Region | | 74 | 3.47 (0.96) | 3.01 (1.13) | 2.46 (0.94) | 3.77 (0.59) | 3.97 (0.64) |
|  | South Western Region | | 82 | 2.63 (0.94) | 2.98 (1.06) | 3.65 (1.31) | 3.13 (1.04) | 3.22 (1.05) |
|  | South Eastern Region | | 80 | 2.94 (0.81) | 3.22 (0.95) | 2.74 (1.37) | 3.16 (0.98) | 3.28 (0.86) |
|  | Pelagonija Region | | 126 | 2.49 (0.89) | 3.37 (0.75) | 3.21 (1.13) | 2.97 (1.00) | 2.86 (0.95) |
|  | Polog Region | | 117 | 2.31 (0.83) | 2.67 (1.00) | 3.96 (0.94) | 3.09 (0.92) | 3.03 (1.04) |
|  | North Eastern Region | | 75 | 2.57 (0.97) | 2.5 (1.11) | 3.73 (1.07) | 2.67 (0.93) | 2.94 (1.04) |
|  | Skopje Region | | 252 | 2.47 (0.84) | 2.77 (1.04) | 3.46 (1.14) | 3.21 (0.83) | 2.92 (1.17) |
| **Serbia** | | | **900** | **2.35 (0.91)** | **2.95 (0.98)** | **3.09 (1.14)** | **3.45 (1.01)** | **3.47 (0.95)** |
|  | Beograd | | 169 | 2.20 (0.96) | 3.29 (0.93) | 2.77 (1.01) | 3.36 (0.91) | 3.87 (0.86) |
| **Serbia** | | |  |  |  |  |  |  |
|  | Vojvodina | | 196 | 2.74 (0.96) | 2.95 (0.96) | 2.77 (1.02) | 3.5 (1.13) | 3.41 (0.98) |
|  | Central Western Serbia | | 322 | 2.12 (0.76) | 2.67 (0.91) | 3.37 (1.17) | 3.52 (0.95) | 3.43 (0.95) |
|  | South Eastern Serbia | | 213 | 2.44 (0.89) | 3.08 (1.03) | 3.23 (1.17) | 3.39 (1.04) | 3.27 (0.89) |

| **Table S7**: Standardized estimates for all DVs controlling for participants’ sex, mobility, engagement, education, experienced discrimination, age, and ethnicity at the individual level and for the urbanity and ethnic fractionalization of the region,including the country at the contextual level | | | | | | | | | | | | | | | |  |
| --- | --- | --- | --- | --- | --- | --- | --- | --- | --- | --- | --- | --- | --- | --- | --- | --- |
|  | **Approach** | | |  | **Social Distance** | | |  | **Forgiveness** | | |  | **Trust** | | |  |
|  | **β (SE)** | ***CI*** | ***p*** |  | **β (SE)** | ***CI*** | ***p*** |  | **β (SE)** | ***CI*** | ***p*** |  | **β (SE)** | ***CI*** | ***p*** |  |
| **Within-level effect** | 0.227 (0.017) | [0.19-0.26] | <.001 |  | -0.188 (0.016) | [-0.22--0.16] | <.001 |  | 0.131 (0.017) | [0.10-0.16] | <.001 |  | 0.1 (0.018) | [0.07-0.13] | <.001 |  |
| **Between-level effect** | 0.844 (0.052) | [0.74-0.95] | <.001 |  | -0.655 (0.094) | [-0.84--0.47] | <.001 |  | 0.71 (0.096) | [0.52-0.90] | <.001 |  | 0.586 (0.119) | [0.35-0.82] | <.001 |  |
| **Contextual effect** | 0.617 (0.055) | [0.51-0.72] | <.001 |  | -0.467 (0.095) | [-0.65--0.28] | <.001 |  | 0.579 (0.097) | [0.39-0.77] | <.001 |  | 0.486 (0.12) | [0.25-0.72] | <.001 |  |
| **Effect size of contextual effect** | 1.24 (0.11) | [1.02-1.46] | <.001 |  | -0.949 (0.193) | [-1.33--0.57] | <.001 |  | 1.162 (0.195) | [0.78-1.54] | <.001 |  | 0.975 (0.241) | [0.50-1.45] | <.001 |  |
| **Contextual effect without controls** | 0.591 (0.063) | [0.47-0.72] | <.001 |  | -0.474 (0.097) | [-0.66--0.28] | <.001 |  | 0.569 (0.097) | [0.38-0.76] | <.001 |  | 0.448 (0.133) | [0.19-0.71] | <.001 |  |
| **Variance decomposition** | | Estimate |  |  |  | Estimate |  |  |  | Estimate |  |  |  | Estimate |  |  |
| **Level 1:** | | 1.000 |  |  |  | 1.000 |  |  |  | 1.000 |  |  |  | 1.000 |  |  |
| **Level 2:** | | 1.000 |  |  |  | 1.000 |  |  |  | 1.000 |  |  |  | 1.000 |  |  |

| **Table S8:** Standardized estimates for all DVs controlling for participants’ sex, mobility, engagement, education, experienced discrimination, age, ethnicity at the individual level and for the urbanity and ethnic fractionalization of the region, including the country at the contextual level for majority and minority groups | | | | | | | | | | | | | | | |  |
| --- | --- | --- | --- | --- | --- | --- | --- | --- | --- | --- | --- | --- | --- | --- | --- | --- |
|  | **Approach** | | | | | | |  | **Social Distance** | | | | | | |  |
|  | **Majority** | | |  | **Minority** | | |  | **Majority** | | |  | **Minority** | | |  |
|  | **β (SE)** | ***CI*** | ***p*** |  | **β (SE)** | ***CI*** | ***p*** |  | **β (SE)** | ***CI*** | ***p*** |  | **β (SE)** | ***CI*** | ***p*** |  |
| **Within-level effect** | 0.206 (0.023) | [0.16-0.25] | <.001 |  | 0.163 (0.03) | [0.11-0.22] | <.001 |  | -0.144 (0.02) | [-0.18--0.10] | <.001 |  | -0.232 (0.026) | [-0.28- -0.18] | <.001 |  |
| **Between-level effect** | 0.634 (0.134) | [0.37-0.90] | <.001 |  | 0.762 (0.089) | [0.59-0.94] | <.001 |  | -0.367 (0.124) | [-0.61--0.12] | 0.003 |  | -0.61 (0.114) | [-0.83- -0.39] | <.001 |  |
| **Contextual effect** | 0.428 (0.128) | [0.18-0.68] | <.001 |  | 0.598 (0.095) | [0.41-0.78] | <.001 |  | -0.223 (0.122) | [-0.46-0.02] | 0.07 |  | -0.377 (0.118) | [-0.61- -0.15] | 0.001 |  |
| **Effect size of contextual effect** | 0.934 (0.249) | [0.45-1.42] | <.001 |  | 1.219 (0.192) | [0.84-1.60] | <.001 |  | -0.496 (0.26) | [-1.00-0.01] | 0.06 |  | -0.765 (0.239) | [-1.23- -0.30] | 0.001 |  |
| **Contextual effect without controls** | 0.57 (0.076) | [0.42-0.72] | <.001 |  | 0.599 (0.101) | [0.40-0.80] | <.001 |  | -0.546 (0.096) | [-0.73--0.36] | <.001 |  | -0.351 (0.133) | [-0.61- -0.09] | 0.01 |  |
| **Variance decomposition** | | Estimate |  |  |  | Estimate |  |  |  | Estimate |  |  |  | Estimate |  |  |
| **Level 1:** | | 1.000 |  |  |  | 1.000 |  |  |  | 1.000 |  |  |  | 1.000 |  |  |
| **Level 2:** | | 1.000 |  |  |  | 1.000 |  |  |  | 1.000 |  |  |  | 1.000 |  |  |

| **Table S8 (continued):** Standardized estimates for all DVs controlling for participants’ sex, mobility, engagement, education, experienced discrimination, age, ethnicity at the individual level and for the urbanity and ethnic fractionalization of the region, including the country at the contextual level for majority and minority groups | | | | | | | | | | | | | | | |  |
| --- | --- | --- | --- | --- | --- | --- | --- | --- | --- | --- | --- | --- | --- | --- | --- | --- |
|  | **Forgiveness** | | | | | | |  | **Trust** | | | | | | |  |
|  | **Majority** | | |  | **Minority** | | |  | **Majority** | | |  | **Minority** | | |  |
|  | **β (SE)** | ***CI*** | ***p*** |  | **β (SE)** | ***CI*** | ***p*** |  | **β (SE)** | ***CI*** | ***p*** |  | **β (SE)** | ***CI*** | ***p*** |  |
| **Within-level effect** | 0.081 (0.02) | [0.04-0.12] | <.001 |  | 0.12 (0.03) | [0.06-0.18] | <.001 |  | 0.076 (0.021) | [0.03-0.12] | <.001 |  | 0.082 (0.031) | [0.02-0.14] | 0.01 |  |
| **Between-level effect** | 0.749 (0.11) | [0.53-0.96] | <.001 |  | 0.394 (0.19) | [0.02-0.77] | 0.04 |  | 0.666 (0.134) | [0.40-0.93] | <.001 |  | 0.359 (0.181) | [0.00-0.71] | 0.05 |  |
| **Contextual effect** | 0.667 (0.109) | [0.45-0.88] | <.001 |  | 0.274 (0.193) | [-0.10-0.65] | 0.16 |  | 0.589 (0.135) | [0.33-0.85] | <.001 |  | 0.277 (0.184) | [-0.08-0.64] | 0.13 |  |
| **Effect size of contextual effect** | 1.494 (0.205) | [1.09-1.89] | <.001 |  | 0.549 (0.387) | [-0.21-1.31] | 0.16 |  | 1.24 (0.261) | [0.73-1.75] | <.001 |  | 0.557 (0.37) | [-0.17-1.28] | 0.13 |  |
| **Contextual effect without controls** | 0.742 (0.07) | [0.60-0.88] | <.001 |  | 0.286 (0.188) | [-0.08-0.65] | 0.13 |  | 0.57 (0.125) | [0.32-0.81] | <.001 |  | 0.32 (0.179) | [-0.03-0.67] | 0.07 |  |
| **Variance decomposition** | | Estimate |  |  |  | Estimate |  |  |  | Estimate |  |  |  | Estimate |  |  |
| **Level 1:** | | 1.000 |  |  |  | 1.000 |  |  |  | 1.000 |  |  |  | 1.000 |  |  |
| **Level 2:** | | 1.000 |  |  |  | 1.000 |  |  |  | 1.000 |  |  |  | 1.000 |  |  |
